# Measles — United States, January 4–April 2, 2015

**Published:** 2015-04-17

**Authors:** Nakia S. Clemmons, Paul A. Gastanaduy, Amy Parker Fiebelkorn, Susan B. Redd, Gregory S. Wallace

**Affiliations:** 1 Division of Viral Diseases, National Center for Immunization and Respiratory Disease, CDC

Measles is a highly contagious, acute viral illness that can lead to complications such as pneumonia, encephalitis, and death. As a result of high 2-dose measles vaccination coverage in the United States and improved control of measles in the World Health Organization’s Region of the Americas, the United States declared measles elimination (defined as interruption of year-round endemic transmission) in 2000 (1). Importations from other countries where measles remains endemic continue to occur, however, which can lead to clusters of measles cases in the United States. To update surveillance data on current measles outbreaks, CDC analyzed cases reported during January 4–April 2, 2015. A total of 159 cases were reported during this period. Over 80% of the cases occurred among persons who were unvaccinated or had unknown vaccination status. Four outbreaks have occurred, with one accounting for 70% of all measles cases this year. The continued risk for importation of measles into the United States and occurrence of measles cases and outbreaks in communities with high proportions of unvaccinated persons highlight the need for sustained, high vaccination coverage across the country.

Confirmed measles cases in the United States are reported by state and local health departments to CDC using a standard case definition (2). A measles case is considered confirmed if it is laboratory-confirmed or meets the clinical case definition (an illness characterized by a generalized rash lasting ≥3 days, a temperature of ≥101°F [≥38.3°C], and cough, coryza, or conjunctivitis) and is linked epidemiologically to a confirmed case. Measles cases are laboratory confirmed if there is detection in serum of measles-specific immunoglobulin M, isolation of measles virus, or detection of measles virus nucleic acid from a clinical specimen. Cases are considered imported if at least some of the exposure period (7–21 days before rash onset) occurred outside the United States and rash occurred within 21 days of entry into the United States, with no known exposure to measles in the United States during that period. Import-associated cases include 1) imported cases, 2) cases that are linked epidemiologically to imported cases, and 3) cases for which an epidemiologic link has not been identified but the viral genotype detected suggests recent importation.* An outbreak of measles is defined as a chain of transmission of three or more linked cases.

During January 4–April 2, 2015, a total of 159 measles cases (in 155 U.S. residents and four foreign visitors) from 18 states and the District of Columbia were reported to CDC (Figure 1). Patients ranged in age from 6 weeks to 70 years; 26 (16%) were aged <12 months, 18 (12%) were aged 1–4 years, 27 (17%) were aged 5–19 years, 58 (36%) were aged 20–39 years, and 30 (19%) were aged ≥40 years. Twenty-two patients (14%) were hospitalized, including five with pneumonia. No other complications and no deaths have been reported.

A total of 111 cases (70%) have been associated with an outbreak that originated in late December 2014 in Disney theme parks in Orange County, California. The source of the initial exposure has not been identified, but measles cases associated with this outbreak have been reported in seven U.S. states, Mexico, and Canada (3). Measles was laboratory confirmed in 101 (91%) of these cases, either by detection of measles-specific IgM or of measles virus RNA. The B3 genotype was identified in specimens from at least 40 patients associated with this outbreak. B3 is a common measles genotype that has been identified in multiple states and countries (4). Other smaller measles outbreaks in 2015 without a link to Disney theme parks have been reported in Illinois (15 cases), Nevada (nine), and Washington (five).

The majority of the 159 patients with reported measles in the 2015 outbreaks were either unvaccinated (71 [45%]) or had unknown vaccination status (60 [38%]); 28 (18%) had received measles vaccine. Among the 68 U.S. residents who had measles and were unvaccinated, 29 (43%) cited philosophical or religious objections to vaccination, 27 (40%) were ineligible because they were too young to receive vaccination (26 patients) or had a medical contraindication (one), three (4%) represented missed opportunities for vaccination, and nine (13%) had other reasons for not being vaccinated (Figure 2).

Of the 159 measles cases, 153 (96%) were import-associated. Ten cases were classified as direct importations, (six among unvaccinated U.S. residents returning from overseas travel, of whom three were aged 6–11 months and age-eligible for vaccination before departure, and four among foreign visitors). Countries associated with direct importations included Azerbaijan, China, Germany, India, Indonesia, Kyrgyzstan, Pakistan, Qatar, Singapore, and United Arab Emirates (one import each).

## Discussion

High population immunity secondary to high measles vaccination coverage has maintained measles elimination in the United States since declaration of elimination in 2000 (5). Worldwide, however, approximately 20 million measles cases occur annually, and importations to the U.S. will continue to place unvaccinated populations at risk for measles. Measles transmission in pockets of unvaccinated persons increases the risk for transmission to vulnerable groups, such as those who cannot be vaccinated because of underlying medical conditions, or infants too young to be vaccinated.

As in previous years, a sizeable proportion of U.S. residents who became infected with measles had an unknown vaccination status (6). This occurred primarily among adults and reflects the lack of immunization data in registries on adults in the United States. Among the U.S.-resident patients who were confirmed as unvaccinated, the numbers who were ineligible for vaccination and who cited philosophical or religious beliefs as the reason they declined vaccination were similar. Exemptions from mandated immunizations have been shown to increase risk for acquiring disease as well as increasing the risk of a disease outbreak at the community level. Exemption rates are higher in jurisdictions where exemption requirements are procedurally easier to meet (7).†

Health care providers should encourage vaccination of all eligible patients who do not have other evidence of measles immunity. Children without contraindications should receive their first dose of measles, mumps, and rubella (MMR) vaccine at age 12–15 months and a second dose at age 4–6 years. Before international travel, infants aged 6–11 months should receive one dose of MMR and children aged 12 months and older should receive two doses of MMR vaccine separated by at least 28 days. Adults born during or after 1957 who are at high risk for measles (i.e., health care personnel, international travelers, and students at postsecondary educational institutions) and who do not have other evidence of measles immunity should also receive 2 doses of MMR vaccine. Other adults without evidence of measles immunity should receive at least 1 dose of MMR vaccine. 1 dose of MMR vaccine administered to those aged ≥12 months is approximately 93% effective at preventing measles and 2 doses approximately 97% effective (8).

Measles should be considered in the differential diagnosis of patients with febrile illness and rash. Patients with clinical symptoms compatible with measles should be asked about recent travel abroad or contact with travelers, and their vaccination status should be verified. Patients with suspected measles should be promptly screened before entering waiting rooms and appropriately isolated (i.e., in an airborne isolation room or, if not available, in a separate room with the door closed), or have their doctor’s office appointments scheduled at the end of the day to prevent exposure of other patients (9). Serology as well as viral specimens should be collected for laboratory testing. Viral genetic sequencing can be used to detect the genotype of the infection, which can be used to suggest the source of an imported virus and track global transmission patterns (10). To assist state and local public health departments with rapid investigation and control efforts to limit the spread of disease, suspected measles cases should be reported to local health departments immediately. State health departments are required to notify cases of measles to CDC within 24 hours of detection.§

Maintenance of high 2-dose MMR vaccine coverage has been crucial in limiting measles spread from importations in the United States. Most measles importations occur when U.S. citizens travel abroad and have not been appropriately vaccinated. Therefore, it is important to encourage timely delivery of measles vaccination for U.S. residents before overseas travel. In addition, early detection of cases and rapid public health response to outbreaks can serve to limit the spread of illness.

What is already known on this topic?

Measles elimination (i.e., interruption of year-round endemic transmission) has been maintained in the United States since 2000. Despite progress in global measles control, measles remains common in many countries of the world, and measles is imported regularly into the United States.

What is added by this report?

During January 4–April 2, 2015, a total of 159 measles cases (in 155 U.S. residents and four foreign visitors) were reported to CDC. Twenty-two patients (14%) were hospitalized, including five with pneumonia. Over 80% of all cases occurred among persons who were unvaccinated or had unknown vaccination status. One outbreak accounted for 70% of all measles cases this year.

What are the implications for public health practice?

Importations of measles into communities with unvaccinated persons can lead to measles cases and outbreaks in the United States. Maintenance of high vaccination coverage, ensuring timely vaccination before travel, and early detection and isolation of cases are key factors to limit importations and the spread of disease.

## Figures and Tables

**FIGURE 1:**
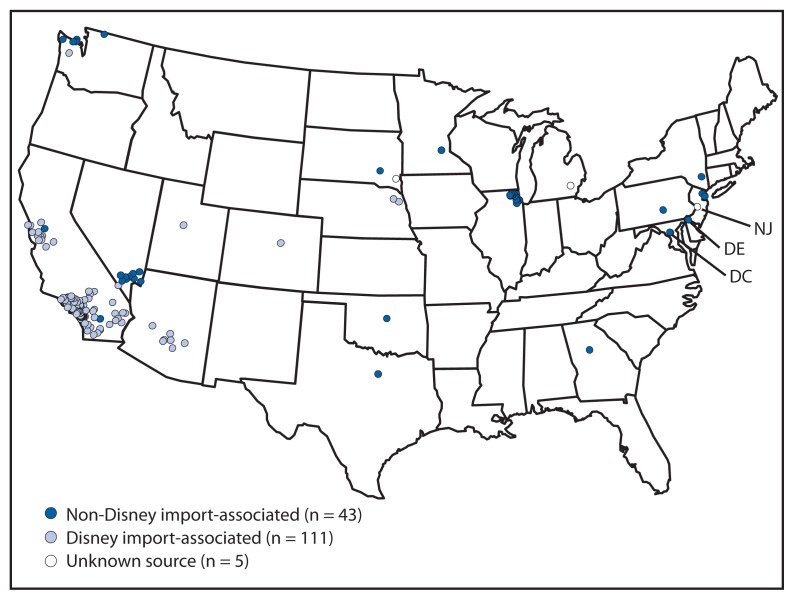
Number of reported measles cases (N = 159), by infection source, state, and county* — United States, January 4–April 2, 2015 **Abbreviations:** DC = District of Columbia; DE = Delaware; NJ = New Jersey. * Cases were reported from 18 states and the District of Columbia, and from 37 counties.

**FIGURE 2:**
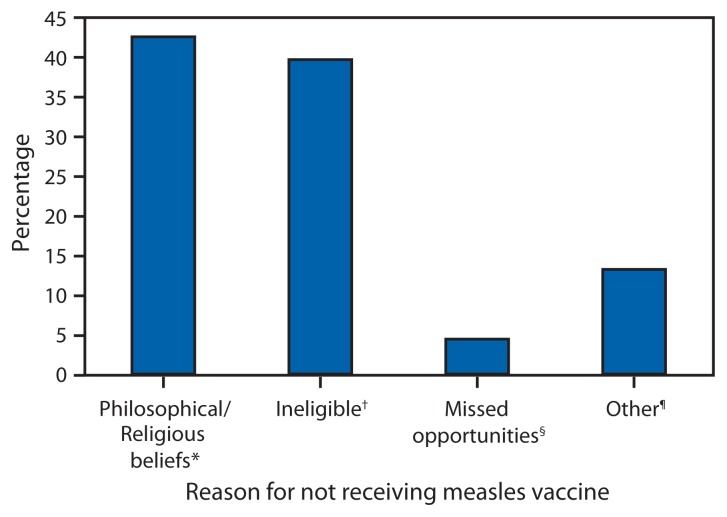
Percentage of U.S. residents with measles who were unvaccinated (n = 68), by reason for not receiving measles vaccine — United States, January 4–April 2, 2015 * Includes persons who were unvaccinated because of their own or a parent’s beliefs. ^†^ Includes persons ineligible for measles vaccination, generally those aged <12 months and those with medical contradictions. ^§^ Includes eligible children aged 16 months–4 years who had not been vaccinated and international travelers aged 6–11 months who were unvaccinated. ^¶^ Includes persons who were known to be unvaccinated and the reason was unknown, and those who were born before 1957 and presumed to be immune.

## Footnotes

* Additional information available at http://www.cdc.gov/vaccines/pubs/surv-manual/chpt07-measles.html.

† Additional information available at http://www.cdc.gov/vaccines/imz-managers/laws/state-reqs.html and at http://www.cdc.gov/phlp/publications/topic/vaccinations.html.

§ Additional information available at http://c.ymcdn.com/sites/www.cste.org/resource/resmgr/pdfs/cstenotifiableconditionlista.pdf.
